# Fibrose Cardíaca e Mudanças Evolutivas na Função Ventricular Esquerda em Pacientes com Cardiopatia Chagásica Crônica

**DOI:** 10.36660/abc.20200597

**Published:** 2021-09-24

**Authors:** João Bosco de Figueiredo Santos, Ilan Gottlieb, Eduardo Marinho Tassi, Gabriel Cordeiro Camargo, Jacob Atié, Sérgio Salles Xavier, Roberto Coury Pedrosa, Roberto Magalhães Saraiva

**Affiliations:** 1 Instituto Nacional de Infectologia Evandro Chagas Fundação Oswaldo Cruz Rio de Janeiro RJ Brasil Instituto Nacional de Infectologia Evandro Chagas/Fundação Oswaldo Cruz, Rio de Janeiro, RJ - Brasil; 2 Casa de Saúde São José Rio de Janeiro RJ Brasil Casa de Saúde São José, Rio de Janeiro, RJ - Brasil; 3 Hospital Universitário Clementino Fraga Filho Faculdade de Medicina Universidade Federal do Rio de Janeiro Rio de Janeiro RJ Brasil Hospital Universitário Clementino Fraga Filho/Faculdade de Medicina/Universidade Federal do Rio de Janeiro, Rio de Janeiro, RJ - Brasil

**Keywords:** Cardiomiopatia Chagásica, Doença de Chagas, Fibrose Endomiocárdica, Disfunção Ventricular Esquerda, Diagnóstico por Imagem, Imagem de Ressonância Magnética/métodos, Eletrocardiografia /métodos

## Abstract

**Fundamento:**

A cardiopatia chagásica (CC) é uma condição de progressão lenta, cujo principal achado histopatológico é fibrose.

**Objetivos:**

Avaliar se a fibrose cardíaca aumenta ao longo do tempo e se correlaciona com aumento no tamanho do ventrículo esquerdo (CE) e redução na fração de ejeção (FE) na CC crônica.

**Métodos:**

Estudo retrospectivo que incluiu 20 indivíduos (50% homens; 60±10 anos) com CC crônica que se submeteram a dois exames de ressonância magnética cardíaca (RMC) com realce tardio com gadolínio em um intervalo mínimo de quatro anos entre os exames. Volume, FE e massa de fibrose do ventrículo esquerdo (VE) foram determinados por RMC. Associações da massa de fibrose na primeira RMC com alterações no volume do VE e FE ventricular esquerda na segunda RMC foram testadas por análise de regressão logística. Valores p<0,05 foram considerados significativos.

**Resultados:**

Os pacientes foram classificados em: A (n=13; alterações típicas de CC no eletrocardiograma e função sistólica global e segmentar do VE normal) e B1 (n=7; alteração na motilidade da parede do VE e FE ≥45%). O tempo médio entre os dois estudos de RMC foi de 5,4±0,5 anos. Fibrose do VE (em % massa do VE) aumentou de 12,6±7.9% para 18,0±14,1% entre os exames de RMC (p=0,02). A massa de fibrose cardíaca no basal associou-se com uma diminuição > cinco unidades absolutas na FE ventricular esquerda da primeira para a segunda RMC (OR 1,48; IC95% 1,03-2,13; p=0,03). A massa de fibrose do VE foi maior e aumentou entre os dois estudos de RMC no grupo de pacientes que apresentaram diminuição na FE entre os testes.

**Conclusões:**

Mesmo pacientes em estágios iniciais da CC apresentam um aumento na fibrose do miocárdio ao longo do tempo, e a presença de fibrose do VE no basal está associada a uma diminuição da função sistólica do VE.

## Introdução

A doença de Chagas é causada pelo protozoário *Trypanosoma cruzi* , que infecta cerca de 10 milhões de pessoas em todo o mundo^1^ e de 1 a 3 milhões de pessoas no Brasil.^2^ Entre os pacientes com doença de Chagas crônica, 20 a 40% apresentam a forma cardíaca da doença – cardiopatia chagásica (CC) –^2^ e cerca de 2% dos pacientes, a cada ano, irão evoluir da forma indeterminada para a forma cardíaca.^3^

Estudos histopatológicos de amostras de miocárdio obtidas de pacientes com CC revelaram uma cardiomiopatia fibrosante crônica leve, com contínua substituição de fibras miocárdicas por áreas de fibrose e hipertrofia compensatória de miócitos remanescentes, o que poderia estar correlacionado com progressão da CC, remodelamento cardíaco e disfunção sistólica do ventrículo esquerdo (VE).^4 , 5^

O exame de ressonância magnética cardíaca (RMC) permite a identificação e a quantificação não invasiva de fibrose cardíaca e de anormalidades da motilidade da parede cardíaca, e a avaliação da função sistólica do VE em pacientes com CC.^6^ A massa de fibrose correlaciona-se diretamente com a classe funcional e inversamente com a fração de ejeção do VE.^7^ Ainda, fibrose identificada por RMC associa-se com arritmias ventriculares,^8^ especialmente na presença de duas ou mais áreas contíguas de fibrose transmural.^9^ Estudos longitudinais relataram que a massa de fibrose foi um preditor independente do desfecho combinado de morte cardiovascular, taquicardia ventricular sustentada,^10^ e mortalidade por todas as causas.^11^

Ainda, a RMC pode identificar áreas de fibrose em aproximadamente 20% dos pacientes com a forma indeterminada de doença de Chagas,^7 , 8^ e em 43,7% dos pacientes no estágio A da CC,^8^ com função sistólica de VE normal (segmentar ou global) na ecocardiografia bidimensional. Por outro lado, em pacientes com estágios mais avançados da forma cardíaca, fibrose cardíaca é detectada em 89%-100% dos pacientes.^7 , 9^ Assim, a RMC pode identificar o envolvimento cardíaco precoce na doença de Chagas e a prevalência de pacientes com fibrose cardíaca aumenta com a gravidade das doenças.

Assim, nosso objetivo foi avaliar se a fibrose cardíaca aumenta ao longo do tempo, e se correlaciona com piora da função e geometria do VE. Nós avaliamos retrospectivamente um grupo de pacientes em estágios iniciais da CC que se submeteram a dois exames de RMC com um intervalo mínimo de quatro anos entre os testes.

## Métodos

### Amostra do estudo

Este foi um estudo retrospectivo que incluiu uma amostra de conveniência composta de pacientes adultos com doença de Chagas crônica acompanhados regularmente no ambulatório de doença de Chagas.

Os critérios para a classificação da doença de Chagas seguiram o consenso brasileiro de doença de Chagas:^2^ forma indeterminada (sem evidência de envolvimento cardíaco), forma cardíaca [evidência de alterações típicas de CC no eletrocardiograma (ECG)], forma digestiva (evidência de megacólon ou megaesôfago), ou forma cardiodigestiva. A forma cardíaca foi classificada em estágio A [sem sintomas de insuficiência cardíaca (IC), com alterações isoladas no ECG], estágio B (sem sintomas de IC com disfunção sistólica segmentar e global do VE; B1: fração de ejeção do VE ≥45%; B2: fração de ejeção do VE <45%), estágio C (IC sintomática), ou estágio D (IC terminal).

Foram incluídos no estudo todos os pacientes com CC em estágio A ou B1 submetidos a dois exames de RMC pela técnica do realce tardio com gadolínio, com fibrose cardíaca detectada na primeira RMC, e teste negativo para doença arterial coronariana no teste ergométrico com esteira no momento basal. A maioria dos pacientes incluídos haviam participado de um estudo anterior.^8^

Dados epidemiológicos e clínicos, incluindo comorbidades, sintomas, ecocardiograma, ECG, Holter 24 horas, e exames de sangue, foram obtidos por análise de prontuários médicos.

### Ressonância magnética cardíaca (RMC)

O primeiro exame de RMC foi realizado em um aparelho GE HDxt de 1,5 Tesla (T) (Wakeusha, Wisconsin, EUA), e analisado utilizando o programa ReportCard^®^ GE, versão 3.6, como descrito anteriormente.^8^ O segundo exame foi realizado em um aparelho Siemens Avanto de 1,5 T (Siemens Healthcare, Alemanha), ou um aparelho Siemens Verio 3,0 T (Siemens Healthcare, Alemanha). As imagens do VE foram obtidas durante pausa respiratória de 15 segundos, a fim de minimizar artefatos causados por movimentos respiratórios. Imagens do eixo longo e do eixo curto do VE foram obtidas por sequências desencadeadas por ECG nos mesmos locais. Análises da função sistólica do VE e do ventrículo direito foram realizadas por cine-RMC, usando o protocolo de imagens de precessão livre em estado estacionário e o diâmetro diastólico do VE, volume diastólico e sistólico do VE, massa ventricular esquerda, fração de ejeção do VE. Volume diastólico do ventrículo direito, e fração de ejeção foram determinados. Os músculos papilares foram considerados como parte da cavidade do VE para o cálculo do volume e da massa do VE. As imagens foram adquiridas a partir de cortes de 8mm e espaçamento de 2mm até o ápice do VE. Utilizou-se uma sequência de pulso gradiente eco com inversão-recuperação para avaliação de realce tardio do miocárdio (RTM) em eixos longo e curto do VE. A presença, local, e padrão da fibrose foram determinados qualitativamente. A massa da fibrose foi calculada usando o programa ReportCard®GE versão 3.6 na primeira RMC, e o programa CVI42 (Circle Cardiovascular Imaging, Canadá) na segunda RMC. O cálculo da massa de fibrose baseou-se na detecção semiautomática de áreas hiperintensas compatíveis com fibrose no eixo curto nas sequências de RTM. O pesquisador tinha liberdade em delimitar a área de fibrose. Foi adotado um limiar de sinal ≥3 desvios padrões (DPs) acima do sinal médio do miocárdio de referência para se determinar o volume de cicatrização para os dois programas utilizados para o cálculo da massa de fibrose. RTM segmentar foi analisado utilizando-se o modelo de divisão do VE em 17 segmentos.^12^ O padrão de cicatrização foi classificado como: 1) transmural, se houvesse qualquer área de cicatriz com ocupação >50% da espessura da parede, em menos de oito segmentos; 2) focal, se a área de cicatrização não fosse transmural e fosse identificada em menos de oito segmentos; e 3) difusa, se as áreas de cicatrização estivessem presentes em mais de oito segmentos, independentemente da presença de áreas transmurais.^10^ Fibrose em segmentos individuais foi classificada como subendocárdica, medial, subepicárdica, ou transmural.

As análises da primeira RMC foi realizada por dois observadores, e as análises da segunda RMC por dois observadores diferentes, que desconheciam os resultados do primeiro exame.

### Análise estatística

Os cálculos foram realizados utilizando o programa estatístico MedCalc 12.5.0.0. As variáveis contínuas foram expressas em média ± DP, e as variáveis categóricas em valores absolutos e porcentagens. Todas as variáveis contínuas passaram o teste de normalidade (teste de KolmogorovSmirnov), permitindo o uso de testes paramétricos. Os dados entre a primeira e a segunda RMC foram comparados pelo teste t de Student pareado. A associação entre a massa de fibrose na primeira RMC e alterações na estrutura e função do VE da primeira para a segunda RMC foram testadas por análise de regressão logística. Uma diminuição superior a cinco unidades na fração de ejeção do VE, um aumento > 10mL/m^2^ no volume diastólico do VE, e um aumento > 10mL/m^2^ no volume sistólico do VE foram considerados eventos nessa análise. Valores p menores que 0,05 foram considerados significativos.

## Resultados

### Características dos pacientes

Vinte pacientes foram incluídos no estudo. Todos apresentavam CC; 65% no estágio A e 35% no estágio B1 da doença na primeira RMC. Doença digestiva associada esteve presente em 35% dos participantes. Observou-se uma igual distribuição de sexos, a maioria dos pacientes nasceu na região nordeste do país, era de etnia branca, apresentava ensino fundamental completo, e era hipertensa ( Tabela 1 ). Nenhum paciente apresentava história de parada cardíaca súbita, sintomas de IC, uso de marcapasso, ou diabetes mellitus. O sintoma mais comum foi palpitações, seguido de pré-síncope, e síncope ( Tabela 1 ). Um paciente teve história de acidente vascular cerebral e outro de ataque isquêmico transitório.

**Tabela 1:** – Características clínicas e epidemiológicas dos participantes do estudo (n=20)

Idade, anos	60,5 ± 10,4
**Sexo masculino**	**10 (50%)**
**Região de origem**	
Nordeste	14 (70%)
Sudeste	5 (25%)
Centro-oeste	1 (5%)
**Etnia**	
Caucasiana	14 (70%)
Mista/Parda	4 (20%)
Afro-brasileira	2 (10%)
**Escolaridade**	
Analfabeto	2 (10%)
Ensino fundamental	12 (60%)
Ensino médio	6 (30%)
**Parâmetros clínicos**	
**Forma clínica da DC**	
Cardíaca – Estágio A	9 (45%)
Cardíaca – Estágio B1	4 (20%)
Cardiodigestiva A	4 (20%)
Cardiodigestiva B1	3 (15%)
**Sintomas**	
Pré-síncope	5 (25%)
Síncope	3 (15%)
Palpitações	7 (35%)
**Hipertensão**	**13 (65%)**
**Eletrocardiograma**	
Bloqueio de ramo direito	14 (70%)
Hemibloqueio anterior esquerdo	14 (70%)
Alterações primárias da onda T	18 (90%)
Contração ventricular prematura	5 (25%)
**Holter 24h Contração ventricular prematura**	
> 30/hora	7 (36,8%)
10-30/hora	3 (15,8%)
< 10/hora	5 (26,3%)
Ausente	4 (21%)
Taquicardia ventricular não sustentada	4 (21%)
**Ecocardiograma**	
Diâmetro atrial esquerdo, cm	3,5 ± 0,5
Diâmetro diastólico do VE, cm	5,1 ± 0,5
Diâmetro sistólico do VE, cm	3,3 ± 0,5
Fração de ejeção do VE, %	64,9 ± 7,9
Aneurisma do VE	2 (10%)
**Função diastólica do VE**	
Normal	10 (50%)
Relaxamento tardio	9 (45%)
Não determinada	1 (5%)

*DC: Doença de Chagas; VE: ventrículo esquerdo; Valores em média ± DP ou n (%).*

Em relação ao ECG, todos os pacientes estavam em ritmo sinusal, e as principais alterações no ECG foram bloqueio de ramo direito, hemibloqueio anterior esquerdo, e alterações primárias da onda T ( Tabela 1 ). Nenhum participante apresentou bloqueio de ramo esquerdo, baixa voltagem do QRS, ou períodos de inatividade elétrica. Com exceção de um participante, todos os pacientes apresentavam resultados de Holter de 24 horas registrados nos prontuários. Nenhum paciente apresentou taquicardia ventricular sustentada, e somente três apresentaram pausa sinusal maior que dois segundos. Quase 40% apresentaram uma elevada incidência de contrações ventriculares prematuras, e um quinto apresentou taquicardia ventricular não sustentada nos exames de Holter ( Tabela 1 ). Com exceção de dois pacientes que apresentavam diâmetro sistólico do VE aumentado, e um paciente com diâmetro diastólico do VE aumentado, todos os participantes apresentaram diâmetros do VE e fração de ejeção normais. Metade dos pacientes também apresentaram função diastólica do VE normal ( Tabela 1 ).

Na ocasião da segunda RMC, três pacientes evoluíram do estágio A para o estágio B1; um paciente progrediu do estágio B1 para B2, e um paciente progrediu do estágio B1 para C. Nenhum paciente apresentou algum evento clínico compatível com síndrome coronária aguda durante o acompanhamento.

### Ressonância magnética cardíaca

**Figura 1:**
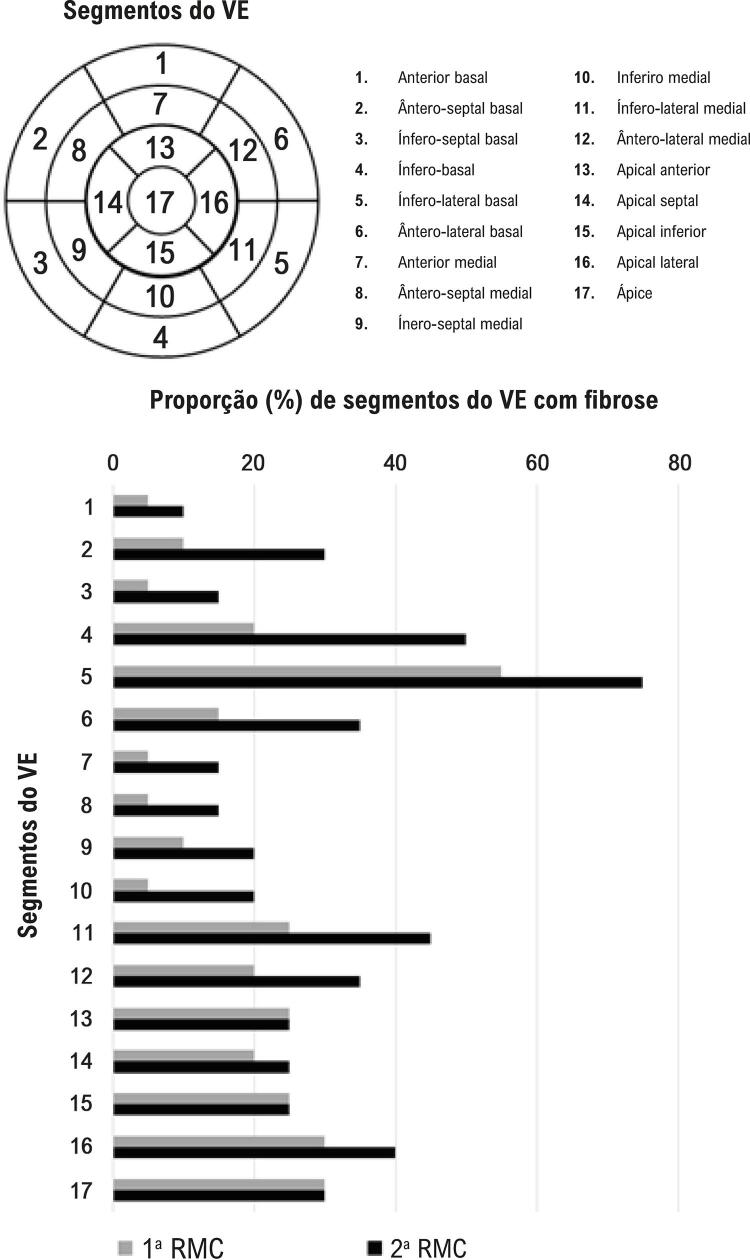
– *Proporção de segmentos do ventrículo esquerdo com fibrose na primeira e na segunda ressonância magnética cardíaca segundo modelo de divisão do ventrículo esquerdo em 17 segmentos.12 Note o aumento na frequência em quase todos os segmentos; VE: ventrículo esquerdo; RMC: ressonância magnética cardíaca.*

O tempo médio entre os exames de RMC foi de 5,4 ± 0,5 anos. A proporção de segmentos do VE com áreas de cicatrização no primeiro e no segundo exames de RMC está apresentada na Figura 1 .

O padrão de fibrose foi classificado como focal em 13 pacientes (65%) e transmural em sete pacientes (35%) na primeira RMC. Sessenta e dois das 340 paredes (18,2%) do VE apresentaram fibrose cardíaca com a distribuição: ínfero-lateral basal (55%), apical (30%), apical lateral (30%), apical inferior (25%), apical anterior (25%), ínfero-lateral medial (25%), ínfero-basal (20%), apical septal (20%), ântero-lateral medial (20%), ântero-lateral basal (15%), ântero-septal basal (10%), ínfero-septal medial (10%), anterior basal (5%), ínfero-septal (5%), anterior medial (5%), inferior medial (5%), e ântero-septal medial (5%). O padrão de fibrose foi classificado como parede medial em 37 segmentos, transmural em 23 segmentos, subepicardial e parede medial em um segmento, e subendocardial e parede medial em um segmento.

Na segunda RMC, o padrão de fibrose apresentado pelos pacientes foi classificado como focal em 13 pacientes (65%), transmural em três pacientes (15%), e difuso em quatro pacientes (20%). O número de paredes com áreas de fibrose aumentou para 102 de 340 paredes (30%) e a frequência de paredes com áreas de fibrose foi: ínfero-lateral basal (75%), inferior basal (50%), ínfero-lateral medial (45%), apical lateral (40%), ântero-lateral basal (35%), ântero-lateral medial (35%), ântero-septal basal (30%), apical (30%), apical inferior (25%), apical anterior (25%), apical septal (25%), ínfero-septal medial (20%), inferior medial (20%), ínfero-septal basal (15%), anterior medial (15%), ântero-septal medial (15%), e anterior basal (10%). O padrão de fibrose foi classificado como mesocárdico em 74 segmentos, transmural em 25 segmentos, mesocárdico e subepicárdico em um segmento, e mesocárdico e subendocárdico em dois segmentos.

Em relação à função e tamanho do VE, os valores médios do volume sistólico do VE e da massa do VE foram maiores, e a fração de ejeção do VE foi menor no segundo exame de RMC em comparação à primeira RMC. O diâmetro diastólico do VE médio, o volume médio do ventrículo direito, e a fração de ejeção do VE média não mudaram significativamente da primeira para a segunda RMC ( Tabela 2 ).

**Tabela 2:** – Comparação da fibrose no ventrículo esquerdo, tamanho e função dos ventrículos direito e esquerdo entre a primeira e a segunda ressonância magnética cardíaca

	1^st^ MRI	2^nd^ MRI	Valor p^a^
Massa de fibrose (g)	12,4 ± 9,1	17,9 ± 16,7	0,03
**Massa de fibrose (% da massa do VE)**	12,6 ± 7,9	18,0 ± 14,1	0,02
**Diâmetro diastólico do VE, mm**	53 ± 4	53 ± 7	0,90
Volume diastólico do VE, mL/m^2^	76,6 ± 19,1	76,8 ± 21,7	0,94
Volume sistólico do VE, mL/m^2^	30,5 ± 13,1	37,9 ± 17,9	0,004
Fração de ejeção do VE, %	61,1 ±9,5	52,5 ± 11,7	<0,0001
**Massa do VE, g/m** ^**2**^	53,9 ± 11,8	56,5 ± 12,6	0,008
Volume diastólico do VD, mL/m^2^	58,9 ± 14,6	62,0 ± 15,8	0,11
Fração de ejeção do VD, %	56,7 ± 3,2	56,1 ± 10,2	0,79

*VE: ventrículo esquerdo; RMC: ressonância magnética cardíaca; VD: ventrículo direito. aTeste t de Student pareado.*

**Figura 2:**
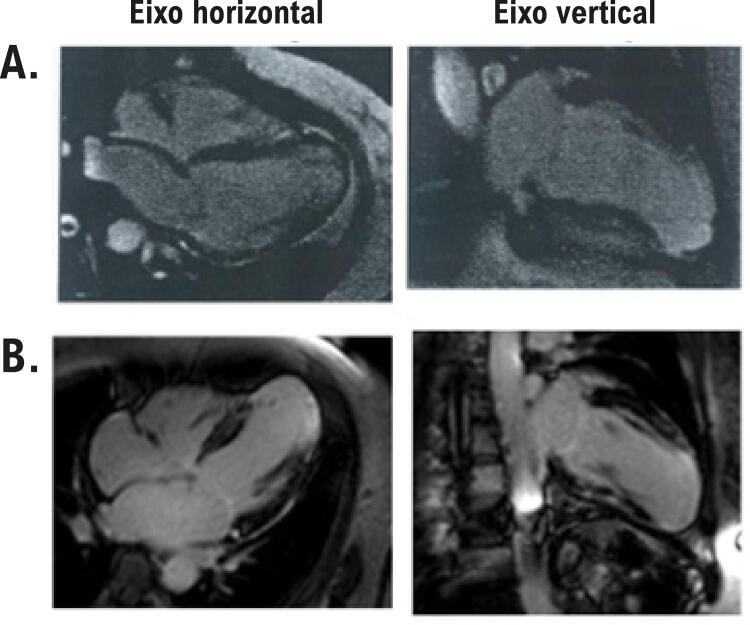
– *Ressonância magnética cardíaca de um paciente com alterações progressivas na massa de fibrose. A) Realce tardio do miocárdio nos cortes do eixo horizontal e vertical mostra áreas de fibrose na parede média (áreas brilhantes) nos segmentos mediais do septo, e fibrose transmural em todas as paredes apicais e no ápice. A massa de fibrose do miocárdio foi estimada em 33 gramas. B) Imagens do realce tardio do miocárdio obtidas do mesmo paciente 4,5 anos depois mostram áreas de fibrose a parede medial nos segmentos basais das paredes ínfero-septal, ínfero-lateral, e ântero-lateral, segmentos mediais das paredes anteriores, e fibrose transmural nos segmentos mediais das paredes ínfero-lateral e ântero-lateral, todas as paredes apicais e ápice. A massa de fibrose do miocárdio foi estimada em 58 gramas.*

**Figura 3:**
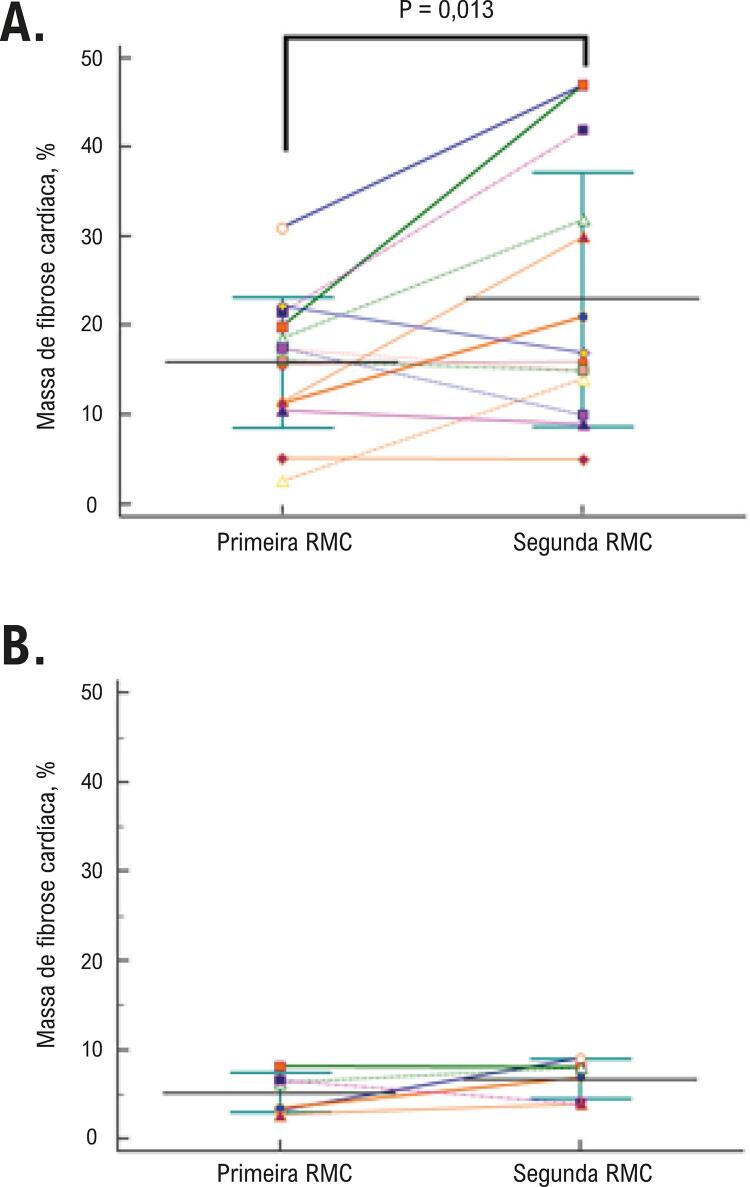
– *Mudanças individuais da massa de fibrose, em porcentagem da massa do ventrículo esquerdo (VE) do primeiro exame de ressonância magnética cardíaca (RMC) ao segundo exame de RMC em pacientes que apresentaram uma diminuição na fração de ejeção do VE ao longo do tempo (A) e pacientes que não apresentaram diminuição na fração de ejeção do VE (B). Note que a massa de fibrose do VE é maior no basal e aumentou da primeira para a segunda RMC somente no grupo de pacientes que apresentaram redução na fração de ejeção do VE ao longo do tempo.*

A massa de fibrose média em porcentagem da massa do VE aumentou 43% da primeira RMC para a segunda RMC ( Figura 2 ; Tabela 2 ). Em relação ao padrão de distribuição da fibrose, os pacientes com um padrão transmural apresentaram aumento na massa de fibrose de 19,3±6.1% a 31,4±14,2%, p=0,02, e aqueles com padrão focal não mostraram aumento significativo na massa de fibrose de 9,0±6,3% a 10,8±7,1%, p=0,36. O número de segmentos do VE com cicatriz aumentou em ambos os grupos: de 38 a 65 (aumento de 71%) no grupo com padrão transmural, e de 24 a 37 (aumento de 54,2%) no grupo com padrão focal de distribuição de fibrose. A massa de fibrose cardíaca aumentou em 11 dos 20 pacientes estudados ( Figura 3 ).

Da primeira para a segunda RMC, 14 pacientes apresentaram uma redução superior a cinco unidades na fração de ejeção do VE, cinco apresentaram um aumento superior a 10mL/m^2^ no volume diastólico do VE e sete apresentaram aumento superior a 10 mL/m2 no volume sistólico final do VE. A massa de fibrose cardíaca em porcentagem da massa ventricular esquerda detectada na primeira RMC mostrou uma associação univariada significativa com uma redução maior que cinco unidades na fração de ejeção do VE (OR 1,48, IC95% 1,03 a 2,13, p=0,03) da primeira para a segunda RMC. Não houve associação significativa univariada ou multivariada entre sexo, idade, massa de fibrose do VE na primeira RMC e mudanças maiores que 10mL/m^2^ no volume diastólico ou sistólico do VE da primeira para a segunda RMC ( Tabela 3 ).

**Tabela 3:** – Associações univariada e multivariada dos eventos estudados com massa de fibrose no ventrículo esquerdo, idade e sexo

	Associação univariada			Associação multivariada		

	OR	IC95%	Valor p	OR	IC95%	Valor p
**Redução > 5 unidades na fração de ejeção do VE**						
Sexo masculino	0,37	0,05-2,77-	0,34	0,14	0,00-15,9	0,42
Idade (anos)	1,03	0,94-1,13	0,47	1,37	0,89-2,11	0,15
Massa de fibrose do VE, %	1,48	1,03-2,13	0,03	2,27	0,87-5,91	0,09
**Aumento >10 mL/m^2^ no volume diastólico do VE**						
Sexo masculino	0,58	0,07-4,56	0,61	0,56	0,04-7,65	0,66
Idade (anos)	1,10	0,96-1,27	0,17	1,12	0,96-1,31	0,15
Massa de fibrose do VE, %	1,04	0,91-1,18	0,56	1,05	0,89-1,24	0,57
**Aumento >10 mL/m^2^ no volume sistólico do VE**						
Sexo masculino	0,64	0,10-4,10	0,64	1,03	0,07-14,8	0,98
Idade (anos)	1,13	0,97-1,31	0,10	1,17	0,97-1,40	0,09
Massa de fibrose do VE, %	1,06	0,94-1,20	0,33	1,11	0,93-1,33	0,24

*VE: ventrículo esquerdo; análise de regressão logística (método Enter).*

Nós estratificamos os pacientes entre aqueles que apresentaram uma diminuição superior a cinco unidades na fração de ejeção do VE e aqueles que não apresentaram ( Figura 3 ). A massa de fibrose do VE, em porcentagem da massa do VE na primeira RMC foi maior entre os pacientes com diminuição na fração de ejeção do VE que nos pacientes que não apresentaram (15,8±7,3% vs. 5,1±2,2%; p=0,017). Ainda, a massa de fibrose do VE em porcentagem da massa do VE aumentou da primeira para a segunda RMC somente nos pacientes que apresentaram uma diminuição na fração de ejeção do VE (15,8±7,3% vs. 22,9±14,2%; p=0,013; Figura 3A ). Entre os pacientes que não apresentaram decréscimo na fração de ejeção do VE a massa de fibrose cardíaca em porcentagem da massa do VE, não se alterou da primeira para a segunda RMC (5,1±2,2% vs. 6,7±2,2%; p=0,25; Figura 3B ).

## Discussão

A CC é uma doença lenta, implacável, silenciosa caracterizada por uma miocardite fibrosante crônica que culmina em uma miríade de eventos cardiovasculares tais como IC, acidente vascular cerebral, e arritmias fatais.^2 , 4 , 5^ Levanta-se a hipótese de que, após um insulto inicial, a lesão cardíaca evolui continuamente até a ocorrência de IC sintomática.^13^ Neste artigo, mostramos, em um grupo de pacientes em estágios iniciais de CC, que a injúria cardíaca causada pela infeção por *T. cruzi* , estimada pela medida massa de fibrose cardíaca, aumenta ao longo do tempo. Além disso, o grau do dano cardíaco, *i.e* ., da massa de fibrose cardíaca, está associado com diminuição da fração de ejeção do VE.

A fibrose cardíaca é uma marca da CC e um índice prognóstico promissor. Até nosso conhecimento, este é o primeiro estudo a abordar mudanças na massa de fibrose cardíaca e estrutura do VE por meio de RMC em pacientes em estágios iniciais da CC. Nós encontramos um aumento não só na massa de fibrose, como no número de segmentos com fibrose, além de uma piora da função sistólica do VE e um aumento no volume sistólico do VE da primeira para a segunda RMC após um período médio de acompanhamento de cinco anos. O aumento na fibrose do VE ocorreu principalmente no grupo de pacientes com fibrose transmural. Em nosso estudo, fibrose do VE foi associada com diminuição da fração de ejeção do VE ao longo do tempo. A massa de fibrose do VE foi maior na primeira RMC e aumentou da primeira para a segunda RMC somente nos pacientes que apresentaram uma diminuição na fração de ejeção do VE.

Pacientes com doença de Chagas com fibrose cardíaca apresentam menor fração de ejeção do VE e maior volume e massa do VE, e maior dimensão do átrio esquerdo que pacientes sem fibrose cardíaca.^10 , 14^ De fato, há uma forte correlação negativa entre massa de fibrose do VE e fração de ejeção do VE.^7 , 15^ Em um estudo anterior de nosso grupo, somente pacientes com fibrose cardíaca apresentaram progressão da doença de Chagas e piora da função do VE medida pela deformação ( *strain* ) longitudinal e circunferencial do VE.^16^ Fibrose do miocárdio associou-se independentemente com mortalidade por todas as causas em um estudo retrospectivo,^11^ e com a ocorrência do desfecho combinado de morte cardiovascular e taquicardia ventricular sustentada em um estudo prospectivo.^10^

Na primeira RMC, fibrose foi mais comumente observada nos segmentos ínfero-lateral e apical, como mostrado anteriormente na doença de Chagas.^7 - 9^ Após um acompanhamento médio de 5,4 anos, a prevalência de fibrose cardíaca aumentou em quase todos os segmentos, mais pronunciadamente nos segmentos basais. Houve uma piora no padrão de fibrose cardíaca, uma vez que, em quatro pacientes, o padrão de fibrose mudou de transmural para difusa. Isso reforça a natureza progressiva do dano cardíaco causado pela doença de Chagas.

Nossos dados mostraram que a massa de fibrose do miocárdio aumenta ao longo do tempo, o que pode estar relacionado com uma miocardite fibrosante de progressão lenta, com alterações na matriz extracelular que leva à remodelação cardíaca e IC. De fato, as enzimas envolvidas na modulação da matriz extracelular [metaloproteinases (MMP) 2 e 9], presentes na patogênese de várias doenças cardiovasculares,^17^ podem ter um importante papel na patogênese da doença de Chagas. Mudanças no equilíbrio entre MMP e suas atividades inibitórias podem ser importantes para remodelação cardíaca.^18^ Em camundongos infectados por *T. cruzi* , tratamento com inibidores de MMP-2/MMP-9 induziu uma redução na inflamação do miocárdio e aumento na sobrevida.^19^ Em pacientes com doença de Chagas crônica, níveis séricos de MMP-2 e MMP-9 são maiores nos pacientes com as formas indeterminada e cardíaca em comparação a controles.^20 , 21^ Contudo, os níveis séricos de MMP-2 foram mais altos em pacientes com a forma cardíaca que a forma indeterminada,^21 , 22^ ao passo que os níveis de MMP-9 foram maiores nos pacientes com a forma indeterminada que a forma cardíaca.^22^ Portanto, um equilíbrio entre MMP-2 e MMP-9 parece ser importante na progressão da doença de Chagas.^23^

### Limitações

Limitações deste estudo incluem seu delineamento retrospectivo, pequena amostra de conveniência, e o fato de os exames de RMC terem sido realizados em equipamentos de diferentes fabricantes, e analisados por diferentes especialistas, sem uma avaliação da variabilidade interobservador. No entanto, a concordância entre os observadores para massa de fibrose cardíaca do grupo que realizou a primeira RMC foi excelente, como já demonstrado previamente.^8^ Em um estudo prévio,^24^ a variabilidade interobservador para a técnica de quantificação de fibrose baseada no limiar de sinal versus miocárdio de referência (do inglês *signal threshold versus reference myocardium* , STRM), que nós usamos para avaliar fibrose do VE, foi -1,2% (IC95% -8,8 a 9,2%).^24^ Em nosso estudo, seis pacientes apresentaram uma diminuição na massa de fibrose no VE em porcentagem da massa do VE, que variou de 1,56 a 7,48%, todos dentro dos limites do intervalo de confiança de 95% para a variabilidade interobservador. Por outro lado, dos 11 pacientes que apresentaram um aumento na massa de fibrose do VE, em porcentagem da massa do VE, sete apresentaram um aumento acima do limite do intervalo de confiança de 95% descrito para a variabilidade interobservador. Três outros pacientes apresentaram uma diferença inferior a um porcento entre os exames. Assim, todos os pacientes com uma diferença entre exames maior que a variabilidade do teste mostraram um aumento na massa de fibrose do VE e corresponderam a 35% da população estudada. Assim, acreditamos que a variabilidade intraobservador e entre observadores não causou viés em nossos resultados.

Quanto à exclusão de pacientes com doença arterial coronariana prévia, um resultado negativo no teste ergométrico com esteira não exclui a possibilidade de uma oclusão total de um ramo coronariano. No entanto, os pacientes negaram a existência de eventos clínicos prévios compatíveis com síndrome coronariana aguda. Ainda, os pacientes não foram submetidos a um segundo teste ergométrico ou à angiografia coronária antes da segunda RMC para excluir possível doença arterial coronariana. Contudo, nenhum paciente apresentou qualquer evento clínico compatível com doença arterial coronariana durante o acompanhamento do estudo.

## Conclusões

Neste estudo retrospectivo que incluiu pacientes em estágios iniciais da CC, a fibrose miocárdica aumentou ao longo do tempo, e a fibrose do VE no basal associou-se a uma diminuição da função sistólica do VE. Esse importante achado deve ser confirmado em estudos prospectivos. A fibrose cardíaca deve ainda ser mais estudada como um índice prognóstico para a evolução da doença de Chagas e eventos cardiovasculares.
