# Supplementary material for: Fibrose Cardíaca e Mudanças Evolutivas na Função Ventricular Esquerda em Pacientes com Cardiopatia Chagásica Crônica
Source: Arq Bras Cardiol. 2021 Sep 24;117(6):1081–90. [Article in Portuguese] doi: 10.36660/abc.20200597 (PMC8757166; doi:10.36660/abc.20200597)
Supplement: Translation

# Translation: Cardiac Fibrosis and Changes in Left Ventricle Function in Patients with Chronic Chagas Heart Disease

**Authors:** João Bosco de Figueiredo Santos, Ilan Gottlieb, Eduardo Marinho Tassi, Gabriel Cordeiro Camargo, Jacob Atié, Sérgio Salles Xavier, Roberto Coury Pedrosa, Roberto Magalhães Saraiva

**Affiliations:** 1 Brazil Instituto Nacional de Infectologia Evandro Chagas/Fundação Oswaldo Cruz, Rio de Janeiro, RJ - Brazil; 2 Brazil Casa de Saúde São José, Rio de Janeiro, RJ - Brazil; 3 Brazil Hospital Universitário Clementino Fraga Filho/Faculdade de Medicina/Universidade Federal do Rio de Janeiro, Rio de Janeiro, RJ - Brazil

**Keywords:** Chagas Cardiomyopathy, Chagas Disease, Endomyocardial Fibrosis, Ventricular Dysfunction, Left, Diagnostic, Imaging, Magnetic Resonance Imaging/methods, Electrocardiography/methods

## Abstract

**Background:**

Chagas heart disease (CHD) is a slow progressing condition with fibrosis as the main histopathological finding.

**Objectives:**

To study if cardiac fibrosis increases over time and correlates with increase in left ventricular (LV) size and reduction of ejection fraction (EF) in chronic CHD.

**Methods:**

Retrospective study that included 20 individuals (50% men; 60±10 years) with chronic CHD who underwent two cardiac magnetic resonance imaging (MRI) with late gadolinium enhancement with a minimum interval of four years between tests. LV volume, EF, and fibrosis mass were determined by cardiac MRI. Associations of fibrosis mass at the first cardiac MRI and changes in LV volume and EF at the second cardiac MRI were tested using logistic regression analysis. P values <0.05 were considered significant.

**Results:**

Patients were classified as follows: A (n=13; changes typical of CHD in the electrocardiogram and normal global and segmental LV systolic function) and B1 (n=7; LV wall motion abnormality and EF≥45%). Mean time between cardiac MRI studies was 5.4±0.5 years. LV fibrosis (in %LV mass) increased from 12.6±7.9% to 18.0±14.1% between MRI studies (p=0.02). Cardiac fibrosis mass at baseline was associated with decrease in >5 absolute units in LV EF from the first to the second MRI (OR 1.48, 95% CI 1.03-2.13, p=0.03). LV fibrosis mass was larger and increased between MRI studies in the group that presented decrease in LV EF between the tests.

**Conclusions:**

Even patients at an initial stage of CHD show an increase in myocardial fibrosis over time, and the presence of LV fibrosis at baseline is associated with a decrease in LV systolic function.

## Introduction

Chagas disease is caused by the protozoan *Trypanosoma cruzi* that infects around 10 million people worldwide^1^ and 1 to 3 million people in Brazil.^2^ Among those patients with chronic Chagas disease, 20 to 40% have the cardiac form or Chagas heart disease (CHD)^2^ and around 2% of patients will progress each year from the indeterminate to the cardiac form.^3^

Histopathological studies of myocardial specimens obtained from patients with CHD revealed a low-grade chronic fibrosing cardiomyopathy with a continuous replacement of myocardial fibers by areas of fibrosis and compensatory hypertrophy of remnant myocytes, which would be correlated to CHD progression, cardiac remodeling and left ventricular (LV) systolic dysfunction.^4 , 5^

Cardiac magnetic resonance imaging (MRI) allows the non-invasive recognition and quantification of cardiac fibrosis, the identification of global and segmental wall motion abnormalities, and aneurysm and intracardiac thrombi, as well as evaluation of LV systolic function in patients with CHD.^6^ Fibrosis mass correlates directly to functional class and inversely to the LV ejection fraction.^7^ Moreover, fibrosis identified by cardiac MRI is associated to ventricular arrhythmias,^8^ especially in the presence of two or more contiguous areas of transmural fibrosis.^9^ Longitudinal studies found that fibrosis mass was an independent predictor of the combined end-point of cardiovascular death, sustained ventricular tachycardia,^10^ and all-cause mortality.^11^

Furthermore, cardiac MRI can identify areas of fibrosis in around 20% of the patients with the indeterminate form of Chagas disease^7 , 8^ and in 43.7% of patients at the stage A of CHD,^8^ who have normal global and segmental LV systolic function on two-dimensional echocardiography. On the other hand, in patients with more advanced stages of the cardiac form, cardiac fibrosis is detected in 89-100% of the patients.^7 , 9^ Therefore, cardiac MRI can identify early cardiac involvement in Chagas disease and the prevalence of patients with cardiac fibrosis increases with the severity of the disease.

Therefore, we aimed to evaluate if cardiac fibrosis progresses over time and if it correlates with worsening of LV function and LV geometry. For that, we retrospectively evaluated a group of patients at early stages of CHD, who underwent two cardiac MRI tests with a minimum interval of four years between them.

## Methods

### Study subjects

This is a retrospective study that included a convenience sample composed of adult patients with chronic Chagas disease regularly followed at the institutional outpatient Chagas disease clinic.

The criteria of Chagas disease classification followed the Brazilian consensus on Chagas disease:^2^ indeterminate form (no evidence of cardiac involvement), cardiac form (evidence of typical CHD changes in the electrocardiogram [ECG]), digestive form (evidence of megacolon or megaesophagus), or cardiodigestive form. Cardiac form was classified into stage A (no symptoms of heart failure [HF] with isolated changes in the ECG), stage B (no HF symptoms with segmental or global LV systolic dysfunction; B1: LV ejection fraction ≥ 45%; B2: LV ejection fraction < 45%), stage C (symptomatic HF), or stage D (end-stage HF).

All patients with CHD at stages A or B1 who underwent two cardiac MRI tests with late gadolinium enhancement (LGE) protocol within a minimum interval of four years, with cardiac fibrosis detected at the first MRI, and negative test for coronary artery disease on treadmill exercise testing at baseline were included in this study. Most patients included in this study were part of a previous study.^8^

Epidemiological and clinical data, including comorbidities, symptoms, echocardiogram, ECG, 24-hour Holter monitoring, and blood tests, were obtained by analysis of medical records.

### Cardiac Magnetic Resonance Imaging (MRI)

The first cardiac MRI was performed in a GE HDxt 1.5 Tesla (T) MRI scanner (Wakeusha, Wisconsin, USA) and analyzed using the ReportCard^®^GE software, version 3.6, as previously described.^8^ The second cardiac MRI was performed using a Siemens Avanto 1.5 T scanner (Siemens Healthcare, Germany) or a Siemens Verio 3.0 T scanner (Siemens Healthcare, Germany). LV images were obtained during a 15-s breath hold to minimize artefacts due to breathing movements. LV long-axis and short-axis images were obtained by two ECG-triggered pulse sequences at the same locations. LV and right ventricular (RV) systolic function were analyzed by cine-CMR using steady-state free precession protocol. End-diastolic LV diameter, end-diastolic and end-systolic LV volumes, LV mass, LV ejection fraction, end-diastolic RV volume and ejection fraction were determined by the Simpson’s method. Papillary muscles were regarded as part of the LV cavity for calculation of LV volume and mass. Images were acquired with 8-mm slice thickness and 2-mm slice spacing up to the LV apex.

In order to evaluate myocardial fibrosis, images were acquired 10 to 20 min after intravenous bolus of 0.1mmol/kg of gadolinium-based contrast (Dotarem^®^, Guerbet, Aulnay Sous Bois, France) using an inversion-recovery prepared gradient-echo sequence for myocardial delayed enhancement (MDE) protocol in the long- and short-axis projections. The presence, location and pattern of fibrosis were qualitatively determined. Fibrosis mass was calculated using the ReportCard^®^GE software version 3.6 in the first cardiac MRI and using the CVI42 software (Circle Cardiovascular Imaging, Canada) in the second cardiac MRI). Calculation of fibrosis mass was based on semi-automatic detection of hyperintense areas compatible with fibrosis on short-axis MDE sequences. The researcher was free to edit the limits of the area of fibrosis. Signal threshold of ≥ 3 standard deviations (SDs) above the mean signal of the reference myocardium was applied to determine the scar volume for both software programs used for fibrosis mass calculation. LV fibrosis mass was defined in absolute values and as percentage of the LV mass. Segmental MDE was analyzed using LV 17-segment model.^12^ Scar distribution patterns were classified as follows: 1) transmural, if there was any area of scar that occupied >50% of the wall thickness but in no more than eight segments; 2) focal, if the scar area was not transmural and identified in no more than eight segments; and 3) diffuse, if the scar areas were present in more than eight segments, regardless of the presence of transmural areas.^10^ Fibrosis in individual segments was classified as subendocardial, midwall, subepicardial, or transmural.

The analyses of the first cardiac MRI were done by two observers, while the analyses of the second cardiac MRI were done by two other different observers who were unaware of the results of the first cardiac MRI.

### Statistical analysis

Calculations were done using statistical software MedCalc 12.5.0.0. Continuous variables were expressed as mean ± SD, and categorical variables as absolute values and percentages. All continuous variables passed the normality test (Kolmogorov-Smirnov test) allowing the use of parametric tests. Data between first and second cardiac MRI were compared using paired Student’s t-test. Associations between fibrosis mass at the first cardiac MRI and changes on LV structure and function between first and second cardiac MRI were tested using logistic regression analysis. A decrease >5 units in LV ejection fraction, an increase >10 mL/m^2^in end-diastolic LV volume, and an increase >10 mL/m^2^in end-systolic LV volume were considered events for this analysis. P values below 0.05 were considered significant.

## Results

### Patients’ characteristics

A total of 20 patients were included in this study. All patients had CHD and 65% were at stage A and 35% at stage B1 of the disease at the time of the first cardiac MRI. Associated digestive disease was present in 35% of the participants. There was an equal gender distribution, most patients were born in the northeast region of Brazil, were white, had elementary schooling, and had hypertension ( Table 1 ). No participant had history of sudden cardiac arrest, HF symptoms, pacemaker, or diabetes mellitus. Most common symptom was palpitations, followed by near-syncope, and syncope ( Table 1 ). One patient had a history of previous stroke and another of transient ischemic attack.

**Table 1:** – Clinical and epidemiological characteristics of study participants (n=20)

Age, years	60.5 ± 10.4
**Male gender**	**10 (50%)**
**Place of origin**	
Northeast	14 (70%)
Southeast	5 (25%)
Central-West	1 (5%)
**Ethnicity**	
Caucasian	14 (70%)
Mixed/Pardo	4 (20%)
Afro-Brazilian	2 (10%)
Schooling	
Illiterate	2 (10%)
Elementary School	12 (60%)
High school	6 (30%)
**Clinical parameters**	
**ChD clinical form**	
Cardiac – Stage A	9 (45%)
Cardiac – Stage B1	4 (20%)
Cardiodigestive A	4 (20%)
Cardiodigestive B1	3 (15%)
**Symptoms**	
Near-syncope	5 (25%)
Syncope	3 (15%)
Palpitations	7 (35%)
Hypertension	13 (65%)
**Electrocardiogram**	
RBBB	14 (70%)
LAHB	14 (70%)
Primary T wave changes	18 (90%)
Premature ventricular contraction	5 (25%)
**24 h Holter Premature ventricular contraction**	
> 30/hour	7 (36.8%)
10-30/hour	3 (15.8%)
< 10/hour	5 (26.3%)
None	4 (21%)
Nonsustained ventricular tachycardia	4 (21%)
**Echocardiogram**	
Left atrial diameter, cm	3.5 ± 0.5
LV end-diastolic diameter, cm	5.1 ± 0.5
LV end-systolic diameter, cm	3.3 ± 0.5
LV ejection fraction, %	64.9 ± 7.9
LV aneurysm	2 (10%)
**LV diastolic function**	
Normal	10 (50%)
Delayed relaxation	9 (45%)
Not determined	1 (5%)

*ChD: Chagas disease; LAHB: left anterior hemiblock; LV: left ventricular; RBBB: right bundle branch block; values are mean ± SD or n (%).*

Regarding ECG, all patients at baseline were in sinus rhythm, and the most common ECG changes were right bundle branch block, left-anterior hemiblock, and primary T wave changes ( Table 1 ). No participant had left bundle branch block, low QRS voltage, or periods of electrical inactivity. Except for one participant, all had 24-hour Holter monitor exams recorded on medical records. No participant had sustained ventricular tachycardia and only three had sinus pause longer than two seconds. Almost 40% had a high incidence of premature ventricular contractions and one fifth had non-sustained ventricular tachycardia on Holter exams ( Table 1 ). Except for two patients with enlarged end-systolic LV diameter and one patient with enlarged LV diastolic diameter, all participants had normal LV diameters and ejection fraction. Half of the patients also presented normal LV diastolic function ( Table 1 ).

At the time of the second cardiac MRI, three patients progressed from stage A to B1, one patient progressed from stage B1 to B2, and one patient progressed from stage B1 to C. No patient presented any clinical event compatible with acute coronary syndrome during the study follow-up.

### Cardiac MRI

The mean time between the cardiac MRI studies was 5.4 ± 0.5 years. The proportion of the LV segments with areas of scar at the first and second cardiac MRI is depicted in Figure 1 .

**Figure 1:** – *Proportion of left ventricular segments with scar at the first and second cardiac magnetic resonance imaging tests according to the 17-segment model.12 Note the increase in the frequency in almost all segments; LV: left ventricular; MRI: magnetic resonance imaging.*

Fibrosis pattern was classified as focal in 13 patients (65%) and transmural in seven patients (35%) at the first cardiac MRI. Sixty-two out of 340 walls (18.2%) showed cardiac fibrosis with the following distribution: basal inferolateral (55%), apex (30%), apical lateral (30%), apical inferior (25%), apical anterior (25%), mid inferolateral (25%), basal inferior (20%), apical septal (20%), mid anterolateral (20%), basal anterolateral (15%), basal anteroseptal (10%), mid inferoseptal (10%), basal anterior (5%), basal inferoseptal (5%), mid anterior (5%), mid inferior (5%), and mid anteroseptal (5%). The fibrosis pattern was classified as midwall in 37 segments, transmural in 23 segments, subepicardial and midwall in one segment, and subendocardial and midwall in one segment.

At the second cardiac MRI, the fibrosis pattern presented by the patients was classified as focal in 13 patients (65%), transmural in three patients (15%), and diffuse in four patients (20%). The number of walls with areas of fibrosis increased to 102 (30% of 340 walls) and the frequency the walls with areas of fibrosis were: basal inferolateral (75%), basal inferior (50%), mid inferolateral (45%), apical lateral (40%), basal anterolateral (35%), mid anterolateral (35%), basal anteroseptal (30%), apex (30%), apical inferior (25%), apical anterior (25%), apical septal (25%), mid inferoseptal (20%), mid inferior (20%), basal inferoseptal (15%),mid anterior (15%), mid anteroseptal (15%), and basal anterior (10%). The fibrosis pattern was classified as midwall in 74 segments, transmural in 25 segments, subepicardial and midwall in one segment, and subendocardial and midwall in two segments.

Regarding LV size and function, mean values of end-systolic LV volume and LV mass were greater, and the LV ejection fraction was lower at the second cardiac MRI compared to the first cardiac MRI. Mean end-diastolic LV diameter and volume, and the right ventricular volume and ejection fraction did not change significantly from the first to the second cardiac MRI ( Table 2 ).

**Table 2:** – Comparison of left ventricular fibrosis and left and right ventricular chamber size and function between the first and the second cardiac magnetic resonance imaging tests

	1^st^ MRI	2^nd^ MRI	p Value^a^
Fibrosis mass (grams)	12.4 ± 9.1	17.9 ± 16.7	0.03
**Fibrosis mass (% of LV mass)**	12.6 ± 7.9	18.0 ± 14.1	0.02
**End-diastolic LV diameter, mm**	53 ± 4	53 ± 7	0.90
End-diastolic LV volume, mL/m^2^	76.6 ± 19.1	76.8 ± 21.7	0.94
End-systolic LV volume, mL/m^2^	30.5 ± 13.1	37.9 ± 17.9	0.004
**LV ejection fraction, %**	61.1 ±9.5	52.5 ± 11.7	<0.0001
LV mass, g/m^2^	53.9 ± 11.8	56.5 ± 12.6	0.008
End-diastolic RV volume, mL/m^2^	58.9 ± 14.6	62.0 ± 15.8	0.11
RV ejection fraction, %	56.7 ± 3.2	56.1 ± 10.2	0.79

*LV: left ventricular; MRI: magnet resonance imaging; RV: right ventricular. aPaired Student’s t-test.*

The mean fibrosis mass in % of LV mass increased 43% from the first cardiac MRI to the second cardiac MRI ( Figure 2 ; Table 2 ). Regarding the pattern of fibrosis distribution, patients with a transmural pattern showed an increase in fibrosis mass from 19.3±6.1% to 31.4±14.2%, p=0.02, and those with focal pattern presented a non-significant increase in fibrosis mass from 9.0±6.3% to 10.8±7.1%, p=0.36. The number of LV segments with scar increased in both groups: from 38 to 65 (a 71% increase) in the group classified as transmural pattern and from 24 to 37 (a 54.2% increase) in the group classified as focal pattern of fibrosis distribution. The cardiac fibrosis mass increased in 11 of the 20 patients studied ( Figure 3 ).

**Figure 2:** – *Cardiac magnetic resonance imaging of a patient with progressive changes in fibrosis mass. A) Myocardial delayed enhancement on horizontal and vertical long-axis slices depicts areas of midwall fibrosis (bright areas) in mid segments of the septum and transmural cardiac fibrosis in all apical walls and apex. Myocardial fibrosis mass was estimated in 33 grams. B) Myocardial delayed enhancement images obtained from the same patient 4.5 years later depict areas of midwall cardiac fibrosis in basal segments of inferoseptal, inferolateral, and anterolateral walls, mid segments of the septum and anterior walls, and transmural cardiac fibrosis in mid segments of inferolateral and anterolateral walls, all apical walls and apex. The estimated cardiac fibrosis mass increased to 58 grams.*

**Figure 3:** – *Individual changes of cardiac fibrosis mass in % of left ventricular (LV) mass from the first cardiac magnetic resonance imaging test (MRI) to the second cardiac MRI among patients who presented a decrease in LV ejection fraction over time (A) and among those who did not present a decrease in LV ejection fraction (B). Note the LV fibrosis mass is larger at baseline and increased from the first to the second cardiac MRI only in the group who showed a decrease in LV ejection fraction over time.*

From the first to the second cardiac MRI, 14 subjects showed a decrease greater than five units in LV ejection fraction, five subjects presented an increase greater than 10 mL/m^2^in end-diastolic LV volume, and seven subjects showed an increase >10 mL/m^2^in end-systolic LV volume. Cardiac fibrosis mass in % of LV mass detected at the first cardiac MRI showed a univariate significant association, with a decrease of >5 units in LV ejection fraction (OR 1.48, 95% CI 1.03 to 2.13, p=0.03) from the first to the second MRI. There was no univariate or multivariate significant association between sex, age, LV fibrosis mass at the first cardiac MRI and the changes in end-diastolic or end-systolic LV volume > 10 mL/m^2^from the first to the second cardiac MRI ( Table 3 ).

**Table 3:** – Univariate and multivariate associations of the studied events with left ventricular fibrosis mass, age and sex

	Univariate Association			Multivariate Association		
	
	OR	95% CI	p values	OR	95% CI	p values
**Decrease >5 units in LV ejection fraction**						
Male sex	0.37	0.05-2.77-	0.34	0.14	0.00-15.9	0.42
Age, years	1.03	0.94-1.13	0.47	1.37	0.89-2.11	0.15
LV fibrosis mass, %	1.48	1.03-2.13	0.03	2.27	0.87-5.91	0.09
**Increase >10 mL/m^2^in end-diastolic LV volume**						
Male sex	0.58	0.07-4.56	0.61	0.56	0.04-7.65	0.66
Age, years	1.10	0.96-1.27	0.17	1.12	0.96-1.31	0.15
LV fibrosis mass, %	1.04	0.91-1.18	0.56	1.05	0.89-1.24	0.57
**Increase >10 mL/m^2^in end-systolic LV vol**						
Sex, male	0.64	0.10-4.10	0.64	1.03	0.07-14.8	0.98
Age, years	1.13	0.97-1.31	0.10	1.17	0.97-1.40	0.09
LV fibrosis mass, %	1.06	0.94-1.20	0.33	1.11	0.93-1.33	0.24

*LV: left ventricular; Vol: volume logistic regression analysis (enter method).*

We stratified the patients into those who had a decrease>5 units in LV ejection fraction and those who did not ( Figure 3 ). LV fibrosis mass in % of LV mass in the first cardiac MRI was greater among those who had a decrease in LV ejection fraction than those who did not (15.8±7.3% vs. 5.1±2.2%; p=0.017). Also, the LV fibrosis mass in % of LV mass increased from the first to the second cardiac MRI only among patients who presented a decrease in LV ejection fraction (15.8±7.3% vs. 22.9±14.2%; p=0.013; Figure 3A ). Among those who did not present a decrease in LV ejection fraction, the cardiac fibrosis mass in % of LV mass did not change from the first to the second cardiac MRI (5.1±2.2% vs. 6.7±2.2%; p=0.25; Figure 3B ).

## Discussion

CHD is a slow, relentless, silent condition characterized by a chronic fibrosing myocarditis that culminates in a myriad of cardiovascular events such as HF, stroke and life threatening arrythmias.^2 , 4 , 5^ It is hypothesized that after an initial insult, cardiac damage progresses continuously until symptomatic HF supervenes.^13^ In this paper, we show in a group of patients at the initial stages of CHD that the cardiac insult caused by *T. cruzi* infection, measured by cardiac fibrosis mass, increases over time. Moreover, the degree of cardiac damage, *i.e* . cardiac fibrosis mass, is associated with decrease in LV ejection fraction.

Cardiac fibrosis is a hallmark of CHD and a promising prognostic index. As far as we know, this is the first study to address changes in cardiac fibrosis mass and LV structure by means of cardiac MRI in patients at an early stage of CHD. We found an increase not only in fibrosis mass, but also in the number of segments with fibrosis, together with a worsening of LV systolic function and an increase in LV end-systolic volume from the first to the second cardiac MRI after a mean follow-up time of five years. The increase in LV fibrosis occurred mainly in the group of patients with transmural fibrosis. In our study, LV fibrosis was associated with decrease in LV ejection fraction over time. LV fibrosis mass was greater in the first cardiac MRI and increased from the first to the second cardiac MRI only in those patients who showed a decrease in LV ejection fraction over time.

Chagas disease patients with cardiac fibrosis have lower LV ejection fraction and higher LV volume and mass, and larger left atrial size than patients without cardiac fibrosis.^10 , 14^ In fact, there is a negative, strong correlation between LV fibrosis mass and LV ejection fraction.^7 , 15^ In a previous study of our group, only patents with cardiac fibrosis had worsening of Chagas disease and LV function measured by LV longitudinal and circumferential strain.^16^ Myocardial fibrosis was independently associated with all-cause mortality in a retrospective study^11^ and with the occurrence of the combined end-point of cardiovascular death and sustained ventricular tachycardia in a prospective study.^10^

At the first cardiac MRI, fibrosis was more commonly seen in the infero-lateral and apical segments, as previously shown in Chagas disease.^7 - 9^ After a mean follow-up of 5.4 years, the prevalence of cardiac fibrosis increased in almost all segments, more notably in the basal segments. There was an aggravation of cardiac fibrosis, as in four patients, fibrosis pattern changed from transmural to diffuse. This reinforces the progressive nature of the cardiac insult caused by Chagas disease.

Our data demonstrated that myocardial fibrosis mass increases over time, which may be related to a slowly progressive fibrosing myopathy with changes in extracellular matrix that leads to cardiac remodeling and HF. In fact, enzymes involved in the extracellular matrix modulation (metalloproteinases [MMP] 2 and 9), present in the pathogenesis of several cardiovascular diseases,^17^ may have an important role in Chagas disease pathogenesis. Changes in the balance between MMP and their inhibitors activity may be important for cardiac remodeling.^18^ In *T.cruzi* infected mice, MMP-2/MMP-9 inhibitors treatment induced a decrease in myocardial inflammation and a survival increase.^19^ In patients with chronic Chagas disease, both MMP-2 and MMP-9 serum levels are higher in patients at the indeterminate and cardiac forms than controls.^20 , 21^ However, MMP-2 serum levels were higher in patients with the cardiac form than with the indeterminate form,^21 , 22^ while MMP-9 serum levels were higher in patients with the indeterminate form than with the cardiac form.^22^ Therefore, a MMP-2/MMP-9 balance seems to be important for Chagas disease progression.^23^

### Limitations

This study is limited by its retrospective design, a small convenience sample, and by the fact that the cardiac MRIs were performed in machines of different vendors and analyzed by different experts, without an assessment of interobserver variability. However, the interobserver agreement for cardiac fibrosis mass of the group that performed the first cardiac MRI was previously described as excellent.^8^ In a previous study,^24^ the interobserver variability for the signal threshold versus reference myocardium (STRM)-based scar quantification technique, which we used to evaluate LV fibrosis, was -1.2% (95% CI -8.8 to 9.2%).^24^ In our study, six patients had a decrease in LV fibrosis mass in % of LV mass, that ranged from 1.56 to 7.48%, all within the 95% CI described for the interobserver variability. On the other hand, from the 11 patients who presented an increase in LV fibrosis mass in % of LV mass, seven showed an increase above the 95% CI described for the interobserver variability. Three other patients had a difference less than one percent between exams. Therefore, all patients with a difference between exams above the test variability showed an increase in LV fibrosis mass and corresponded to 35% of the studied population. Therefore, we believe that our results were not biased by intra or inter-observer variability.

Regarding exclusion of patients with previous coronary artery disease, a negative treadmill exercise test result does not exclude the possibility of complete occlusion of a coronary branch. However, patients denied previous clinical events compatible with acute coronary syndrome. Patients also did not undergo a second treadmill exercise test or coronary angiography before the second MRI to exclude coronary artery disease. However, no patient presented any clinical event compatible with coronary artery disease during the study follow-up.

## Conclusions

In this retrospective study that included patients at an initial stage of CHD, myocardial fibrosis increased over time and LV fibrosis at baseline was associated with a decrease in LV systolic function. This important finding should be confirmed in prospective designed studies. Cardiac fibrosis must also be further studied as a prognostic index for Chagas disease progression and cardiovascular events.
